# Dr. Hullihen's Method of Mounting Single Teeth

**Published:** 1853-04

**Authors:** 


*Dr. Hullihen's Method of Mounting Single Teeth.*—We had intended to have given a description of Dr. Hullihen's method of mounting single teeth in the present number of the Journal, but not being able to have an illustration prepared in time, we are compelled to defer it until our July issue.

